# The Impact of Whole Body Vibration Therapy on Spasticity and Disability of the Patients with Poststroke Hemiplegia

**DOI:** 10.1155/2018/8637573

**Published:** 2018-05-02

**Authors:** Alev Alp, Bilge Efe, Mihriban Adalı, Adnan Bilgiç, Sevda Demir Türe, Şeyma Coşkun, Merve Karabulut, Uğur Ertem, Selim Mahmut Günay

**Affiliations:** Faculty of Medicine, Physical Therapy and Rehabilitation Department, Uludag University, Bursa, Turkey

## Abstract

**Objective:**

To determine if whole body vibration therapy (WBV) effectively improves functional outcome in patients with poststroke hemiplegia.

**Materials and Methods:**

In this single-blind RCT, WBV group (*n* = 10) had 40 hz frequency/4 mm amplitude vibration during 5 minutes/session, 3 days a week, for a duration of 4 weeks. The control group (*n* = 11) had no vibration therapy for the same duration while standing on the same platform. Patients in both of the groups did 15 minutes of stretching and active range of motion exercises before the intervention. Outcome measures were Modified Ashworth Scale (MAS), Functional Independence Measurement (FIM), and Timed 10-Meter Walk Test (10 mWT).

**Results:**

Only 10 mWT improved at the 1st week (*p* = 0.002), 1st month (*p* < 0.001), and 3rd month (*p* < 0.001) in favor of the intervention group. There was positive correlation also between 10 mWT and ankle spasticity (*p* < 0.001, *r* = 0.931).

**Conclusion:**

This study suggests that WBV therapy may be a complementary therapy in gait rehabilitation and functional outcome of the patients with calf muscle spasticity.

## 1. Introduction

Stroke is a leading cause of functional impairments and spasticity is a major disabling and common symptom in neurologic conditions such as poststroke hemiplegia. It is a velocity dependent increase in tonic stretch reflexes and tendon jerks because of abnormal supraspinal discharges. Spastic movement disorders during walking is a complex problem because it has many components associated with qualitative changes in muscle mechanical properties, such as weakness and increased muscle stiffness influencing walking speed and function [1].

Management strategies include pharmacological agents, physical agents, and exercise to cope with this physical burden. The physical management strategies for reducing spasticity and impairment are briefly muscle stretching, muscle strengthening, shock wave therapy, ultrasound therapy, cryotherapy, electrical stimulation, and vibration [1]. Whole body vibration (WBV) procedure is found to be useful in reducing leg muscle spasticity in cerebral palsy but there is insufficient evidence to support using it in poststroke spasticity [2].

WBV is assumed to apply a low amplitude low frequency (between 2–4 mm and 30–50 Hz, resp.) vibratory stimulus using a mechanical device and generate Ia inputs by activating muscle spindles [3]. These Ia inputs can alter excitability of the corticospinal pathway by modulating fascilatory signaling [3, 4]. These results are believed to initiate muscle contractions by stimulating muscle spindles and alpha motor neurons resulting in an effect similar to physical exercise therefore it is incorporated as a strength training method [5, 6].

Mechanical oscillation defined by amplitude and frequency generates a force that acts on the whole body. It is assumed also that low amplitude WBV can be used for proprioceptive and balance training [7, 8] by means of improving neuromuscular function. Previous studies have demonstrated the functional benefits of WBV in hemiplegic stroke [9, 10].

Therefore, present paper is about the therapeutic effects of WBV combined with exercise. The intervention is supposed to highlight neurophysiological and mechanical effects of vibration on spasticity, functional impairment, and walking speed of the patients with poststroke hemiplegia.

## 2. Materials and Methods

In this single-blind randomized controlled study, 51 patients with poststroke hemiplegia were assessed for eligibility with their cranial MRIs and neurological and locomotor system examinations. 24 patients were enrolled in the study who met the inclusion criteria. Inclusion criteria were as follows: (1) having chronic phase (at least 12 months) unilateral stroke, (2) having an ischemic or hemorrhagic poststroke hemiplegia, (3) having calf muscle spasticity score; 1^+^ to 3 by Modified Ashworth Scale (MAS) [11], and (4) being able to reach at least Brunnstrom stage 3 at the leg. Exclusion criteria were the presence of inflammatory rheumatic or infectious diseases, cardiorespiratory disorders, psychiatric diseases, new fractures, and severe degenerative joint diseases. 2 of the patients met the exclusion criteria; therefore 22 patients (2 females, 20 males) were randomized into 2 groups which were exercise + WBV (*n* = 11) and exercise + no vibration (*n* = 11) groups. Before randomization, the University Hospital Ethical Community Approval was obtained and the subjects were recruited from the outpatient unit of our physical therapy and rehabilitation department and provided informed written consent.

All of the participants did stretching and active range of motion exercises by the supervision of a physiotherapist on the hemiplegic lower extremity for 15 minutes before going on the device. Afterwards, in the intervention group (*n* = 11), WBV of 40 hz frequency/4 mm amplitude was applied for 5 minutes/session, 3 days/week, and for a duration of 4 weeks. The control group (*n* = 11) had no vibration for the same duration and session frequency, on the same platform. In order to have an isometric contraction of the calf muscles, participants were asked to stand still on tiptoes while knees are in 30 degrees semiflexion for 3 × 10 sec (totally 30 sec) consecutively with 3-second intervals. Because this exercise was very difficult for some patients, it was followed by a recovery period of 20 seconds between repetitions. The whole procedure was supervised by a trained physiotherapist.

The device for controlled whole body vibration is called “Compex Winplate” and it is intended solely for vibration training. It has been manufactured by Uniphy Elektromedizin GmbH and CoKG according to international quality standards of the ISO 9001:2000 and DIN EN ISO 13485:2003. It is a round platform with supporting bars for holding with both hands during the tasks. The platform produces vibrations and the intensity is chosen based on previous literature [1–10]. The WBV training of 4 mm amplitude and 40 hz frequency was administered on the calf muscles by the physiotherapist.

In this single-blind RCT, the randomization was done by a computer generated table of random numbers and the assessor (physiatrist) was blind to group allocation and the evaluation of the patients. The patients were advised to have a stable medical condition and drug administration for the duration.

Baseline demographic characteristics assessed were age, sex, duration of illness, dominant hemisphere, the impaired side of the body, and ankle spasticity. Outcome measures of the study were MAS [11], Functional Independence Measurement (FIM) [12], and Timed 10-Meter Walk Test (10 mWT) as seconds [13], which were evaluated at baseline, just after the first session, after 1 and 4 weeks of training (1 month) also after 3 and 6 months. The acute response to vibration training was assessed immediately after the first session and the late response to a potential synaptic modulation was assessed after 6 months, as a rationale. The Functional Independence Measurement (FIM) is widely used and accepted as an assessment tool that evaluates the functional status of patients throughout the rehabilitation process in patients with stroke or other neurologic disorders. There is also evidence existing that FIM scores can be used as an accurate predictor of outcomes in poststroke patients [14]. The ability to quantify the functional ability and motor control of the poststroke patients engaged in a rehabilitation program may assist in prediction of their final functional outcome. Gait disorders are common in this type of patients compromising quality of life and increasing the risk for falls. Therefore, 10-Meter Walk Test is recommended to obtain the most valid clinical assessment of walking speed when using it as a time indicator of health status. Individuals walks without assistance for 10 meters, with the time measured for the intermediate 6 meters allowing for acceleration and deceleration. Assistive devices may be used but must be kept consistent and documented for each test. The spasticity was measured using MAS score for the gastrocnemius muscle group which is a reliable and valid instrument using a 6-point ordinal scale (0, 1, 1 +, 2, 3, 4) to score the average resistance to passive movement for ankle joint. In a recent review of 17 studies, the Modified Ashworth Scale was determined to be the most commonly used clinical measure of lower limb spasticity [15].

### 2.1. Data Analysis

The normality of data between groups was evaluated with Shapiro-Wilk test and categorical variables, such as age characteristics, were compared with independent *t*-test and the uncategorical variables were compared with Mann–Whitney *U* test. Wilcoxon's rank-sum test was used to determine the changes between baseline and followup in each group. Fisher's exact test was used for comparing categorical data and descriptive statistics between outcome measures were defined as “mean ± standard deviation” for categorical variables and as “median (minimum-maximum)” for continuous variables. Relationships between scores were evaluated with Spearman Correlation Analysis. The level of significance for all tests was *α* < 0.05. Data analysis was done by IBM SPSS Statistics 21 programme. Sample size calculation is done by one sample *t*-test power analysis.

## 3. Results

**Figure 1:**
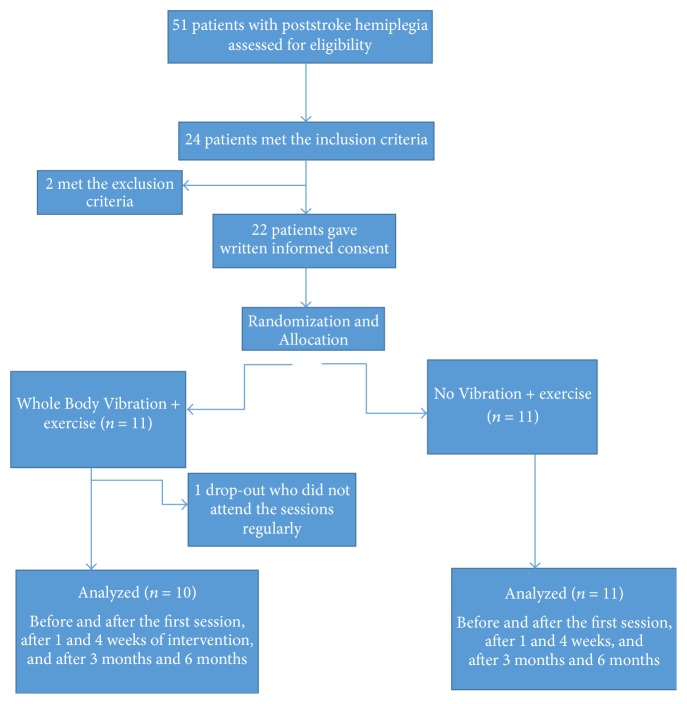
Patients flow diagram.

24 poststroke hemiplegic patients participated in the study from the outpatient clinic. 2 of them met the exclusion criteria and 1 patient was dropout who did not participate in the vibration + exercise sessions properly. Therefore, 21 patients were included in the analysis of whom completed the 4 weeks of intervention (Figure 1).

The mean ages of the WBV (*n* = 10) and control (*n* = 11) groups were 61.2 ± 11 and 62.9 ± 8 years, respectively, and demographic variables were homogeneous except ankle spasticity (*p* < 0.05) and 10 mWT (*p* < 0.001) in favor of the control group (Table 1).

When the groups were compared with each other, no significant difference was found between the variables except 10 mWT at the 1st week (*p* = 0.002), 1st month (*p* < 0.001), and 3rd month (*p* < 0.001) evaluation in favor of the intervention group (Table 2).

There was negative correlation between 10 mWT and FIM (*p* < 0.030, *r* = −0.473) and positive correlation between 10 mWT and ankle spasticity by MAS (*p* < 0.001, *r* = 0.931) at the first week of vibration. Changes in spasticity and walking speed were positively correlated also at the 1st (*p* < 0.001, *r* = 0.881), 3rd (*p* < 0.001, *r* = 0.941), and 6th (*p* < 0.001, *r* = 0.877) month of evaluations (Table 3).

According to the sample size calculation, the difference between the mean values was 14.2 ± 8 standard deviations with the power of 80% for 10 mWT (*α* = 0.05).

## 4. Discussion

The present paper evaluated and found that WBV training combined with isometric exercise had benefits in walking performance on 10-poststroke patients. This result was in positive correlation with reduction in spasticity. The change in motor function assessed by walking speed showed positive correlation with spasticity reduction throughout the study. However the two outcome measures, baseline ankle spasticity and 10 mWT, were in favor of the control group. Because groups were compared with difference scores from baseline, this was neither a disadvantage nor an advantage for the intervention group to be considered important. Moreover, FIM and walking speed were in negative correlation at the first week evaluation. These results may be the consequences of supraspinal effects of vibration by inhibitory pathways or proprioceptive insensitivity of the muscle spindles or solely the enhancement of the calf muscle strength.

This structural adaptation is presumed to be initiated by tonic vibration reflex stimulation which activates muscle receptors in charge of detecting the change in length of a muscle. As another mechanism, vibration training has a potential to induce proprioceptive reflex and counteract the sensation of pain [16]. Vibration training also seems to elicit a warmup effect improving muscle power and balance in subpopulations prone to fall [8].

Recent studies [9, 10, 17–19] also found remarkable benefits of vibration on poststroke spasticity and gait performance though the rest of the vibration studies did not [2, 20–22]. Lukashevich and Likhachev [17] studied focal vibration for its ability to reduce poststroke spasticity in 13 volunteers and found that 80 hz and 0.3 amplitude focal vibrations on gastrocnemius muscle reduce maximum amplitudes of H-reflex and M-response as the reflectors of calf muscle spasticity. Another study [18] about the upper extremity function, spasticity, and grip strength in subjects with poststroke hemiplegia indicated that the use of whole body vibration training combined with task-related training improves arm function, strength, and spasticity. The intervention group had more benefits after 4 weeks of training on the mentioned issues than the control group and the WBV group alone also in accordance with our study. Lee et al. [19] found also short-term (after 30 minutes) improvement in Ashworth scores, F-wave, and motor function of the hand in poststroke patients after treatment.

Despite these studies above, evidence found in stroke is inconsistent. In two recent reviews [21, 22], 4 poststroke vibration studies were evaluated and only one study by Huang et al. [2] demonstrated significant reduction in poststroke spasticity by 8 weeks of vibration training (level Ib). Rest of the 3 stroke trials did not show any significant benefit of vibration training. Undefined subject characteristics and limited methodological and reporting quality in WBV studies are found to be responsible for the insufficient evidence to support or rebut the effects of the procedure. It is also concluded that there is little evidence that “change in spasticity is due to improvement in functional performance” [21].

Chan et al. [23] found improvement in balance and mobility accompanied by reduction in poststroke spasticity after WBV but correlation analysis was not performed. Only Cheng et al. [24] reported that the “time up and go” score was negatively correlated with the change in pendulum test in children with cerebral palsy. The ROM and walking speed were poorly reported in clinical assessment of lower limb spasticity, making it difficult to draw conclusions regarding the relevance of MAS to walking performance [25].

The current study gives positive feedback to some of these literatures but there are some limitations to mention which are the lack of balance testing and using MAS for spasticity assessment tool. The reason is that it is based on the evaluation of increase in muscle tone during resting; therefore, spasticity assessment during functional movements are not taken into account. The spasticity measurement (MAS) was always done by the same physiotherapist in order to overcome the handicap of manual testing.

Evidence suggests that vibration therapy with isometric exercise seems to be a safe complementary or alternative therapy which improves walking speed in relevance with nonsignificant reduction in poststroke spasticity. This receptor activation is assumed to induce the improvement of complete stretching reflex arc. Further research with standardised procedures is needed to conclude vibration training. Clinical scales of spasticity also must be validated in order to understand better the impact of spasticity on functional activities such as walking.

## Figures and Tables

**Table 1:** Baseline evaluation and demographic characteristics of the patients in comparison.

	WBV (*n* = 10)	Control (*n* = 11)	*p*
Age	61.20 ± 11.043	62.91 ± 8.154	0.689
Sex	10 M, 0 F	9 M, 2 F	0.476
Duration of illness (years)	1.5 (1–4)	2 (0.5–5)	0.468
Dominant extremity	8 right, 2 left	10 right, 1 left	0.586
Affected body side	6 right, 4 left	8 right, 3 left	0.659
Ankle spasticity by MAS	3 (2-3)	2 (1–3)	**0.020** ^**∗**^
FIM	106 (103–110)	109 (101–120)	0.863
10 mWT (seconds)	26.9 (18–52.11)	15.23 (11.73–18.05)	<**0.001**^**∗∗∗**^

Values are mean ± standard deviation for categorical variables and median (minimum-maximum) for uncategorical variables; WBV: whole body vibration; MAS: Modified Ashworth Scale; FIM: Functional Independence Measurement; 10 mWT: Timed 10-Meter Walk Test; ^*∗*^*p* < 0.05; ^*∗∗∗*^*p* < 0.001.

**Table 2:** Statistical significance of the percent of changes and difference scores from baseline and group comparisons for these variables.

	WBW group (*n* = 10)	Exercise group (*n* = 11)	*p*
1st session			
Ankle-MAS	0 (0-0)	0 (−1-0)	0.756
FIM	0.009 (0–0.06)	0 (0–0.04)	0.072
10 mWT	−0.124 (−0.17–(−0.07))	−0.079 (−0.15–0.02)	0.197
1st week			
Ankle-MAS	0 (−1-0)	0 (−1-0)	0.973
FIM	0.032 (0–0.08)	0.036 (0–0.07)	0.282
10 mWT	−0.15 (−0.36–(−0.9))	−0.118 (−0.12–0)	**0.002** ^**∗∗**^
1st month			
Ankle-MAS	−0.50 (−1-0)	0 (−1-0)	0.143
FIM	0.039 ± 0.271	0.032 ± 0.023	0.563
10 mWT	−0.253 (−0.37–(−0.16))	−0.093 (−0.25–0)	**0.001** ^**∗∗**^
3rd month			
Ankle-MAS	−1 (−1-0)	0 (−1-0)	0.063
FIM	0.045 ± 0.035	0.065 ± 0.044	0.278
10 mWT	−0.297 (−0.61–(−0.16))	−0.080 (−0.26–(−0.01))	**0.001** ^**∗∗**^
6th month			
Ankle-MAS	−0.50 (−1-0)	0 (0-0)	0.063
FIM	0.063 ± 0.041	0.06 ± 0.044	0.900
10 mWT	−0.219 (−0.43–(−0.02))	−0.093 (−0.27–0)	0.075

Values are mean ± standard deviation for categorical variables and median (minimum-maximum) for uncategorical variables; WBV: whole body vibration; MAS: Modified Ashworth Scale; FIM: Functional Independence Measurement; 10 mWT: Timed 10-Meter Walk Test; ^*∗∗*^*p* < 0.01.

**Table 3:** Correlations between 10 mWT in the intervention group.

	1st week 10 mWT	1st month 10 mWT	3rd month 10 mWT	6th month 10 mWT
1st week				
FIM	*p* = 0.030, *r* = −0.473			
Ankle spasticity by MAS	*p* < 0.001, *r* = 0,931			
1st month				
Ankle spasticity by MAS		*p* < 0.001, *r* = 0.881		
3rd month				
Ankle spasticity by MAS			*p* < 0.001, *r* = 0.941	
6th month				
Ankle spasticity by MAS				*p* < 0.001, *r* = 0.877

MAS: Modified Ashworth Scale, FIM: Functional Independence Measurement, 10 mWT: Timed 10-Meter Walk Test, statistical significance = *p* < 0.05, and *r* = correlation coefficient.
